# Study of Highly Sensitive Formaldehyde Sensors Based on ZnO/CuO Heterostructure via the Sol-Gel Method

**DOI:** 10.3390/s21144685

**Published:** 2021-07-08

**Authors:** Jing Liu, Yan Chen, Hongyan Zhang

**Affiliations:** 1 Xinjiang Key Laboratory of Solid State Physics and Devices, Xinjiang University, Urumqi 830046, China; xdlj@xju.edu.cn (J.L.); cycy1yan@163.com (Y.C.); 2 School of Physical Science and Technology, Xinjiang University, Urumqi 830046, China

**Keywords:** formaldehyde sensor, ZnO/CuO heterostructure, amino groups, hydroxyl groups

## Abstract

Formaldehyde (HCHO) gas sensors with high performance based on the ZnO/CuO heterostructure (ZC) were designed, and the sensing mechanism was explored. FTIR results show that more OH^−^ and N–H groups appeared on the surface of ZC with an increase in Cu content. XPS results show that ZC has more free oxygen radicals (O*) on its surface compared with ZnO, which will react with more absorbed HCHO molecules to form CO_2_, H_2_O and, electrons, accelerating the oxidation-reduction reaction to enhance the sensitivity of the ZC sensor. Furthermore, electrons move from ZnO to CuO in the ZC heterostructure due to the higher Fermi level of ZnO, and holes move from CuO to ZnO until the Fermi level reaches an equilibrium, which means the ZC heterostructure facilitates more free electrons existing on the surface of ZC. Sensing tests show that ZC has a low detection limit (0.079 ppm), a fast response/recovery time (1.78/2.90 s), and excellent selectivity and sensitivity for HCHO detection at room temperature. In addition, ambient humidity has little effect on the ZC gas sensor. All results indicate that the performance of the ZnO sensor for HCHO detection can be improved effectively by ZC heterojunction.

## 1. Introduction

ZnO is an n-type metal oxide semiconductor material of the Ⅱ-Ⅵ groups with high exciton binding energy (60 meV) and a wide direct band gap (3.4 eV), which has attracted a lot of attention in the field of gas sensors due to its rich morphology and excellent chemical and thermal stability [1,2,3,4,5]. However, there are still some disadvantages in the reported ZnO gas sensors, including poor linearity, slow response/recovery time, and low selectivity [6,7]. The performance of ZnO gas sensors can be improved by doping [3,8], hybridization [9,10], surface modification [11,12], and heterostructure [13,14,15,16]. Among these methods, forming a heterostructure with another material is widely considered to be an effective way to improve the performance of gas sensing based on ZnO. For example, Z. Song et al. reported the p-n heterostructure of NiO nanoparticles on ZnO structures to detect acetylene [17], and C. Han et al. reported p-CuO/n-ZnO nanofibers to detect H_2_S gas [18]. These reports indicate that the formation of a p-n heterostructure expands the electron depletion layer on the surface of the material and inhibits the recombination of electrons and holes.

CuO as a p-type metal oxide semiconductor with a band gap of 1.2 eV is usually used for sensors, because of its good thermal stability and high catalytic activity, etc. [1,19,20,21,22] Both the band structure and the difference in charge carrier type between CuO and ZnO are well matched to form a stable p-n heterojunction [23]. It has been reported that ZnO/CuO heterostructure (ZC) sensors can improve the sensing performance and result in the formation of a p-n heterojunction [17,18]. Firstly, the heterojunction structure leads to an increase in active sites of the ZC sensor and expand the electron depletion region, resulting in an increase in electron mobility. Secondly, oxygen can be decomposed rapidly at lower temperatures through the synergistic effect of ZnO and CuO. Recently, ZnO/CuO heterostructure (ZC) materials are used in HCHO detection [24,25,26]. HCHO is a kind of irritating gas, which is seriously dangerous to human health and is considered to be a harmful indoor pollution gas [27,28,29]. Therefore, it is extremely urgent to study HCHO sensors with high performance at room temperature. Furthermore, some literature reported that the presence of functional groups (carboxyl, hydroxyl, amino, carbonyl groups, etc.) on the material’s surface can improve the performance of gas sensors. For example, a copper (II) complex functionalized quartz crystal microbalance (QCM) gas sensor with amino groups was reported by Wang et al. [30], the flexible cotton fiber/polyaniline sensor with hydroxyl groups was reported by Zhang et al. [31], the gold nanoparticles (Au-NPs)/reduced graphene oxide (rGO) sensor functionalized using carboxyl (COOH) was reported by Xia et al. [32], the COOH groups on surface of graphene was reported by You et al. [33], and the Hydrogen adsorption of Mg-Doped Graphene Oxide was reported by Chen et al. [34]. However, HCHO based on a functional group modifying ZC heterostructure has only been reported in a few papers.

In this paper, HCHO gas sensors with high performance based on ZnO/CuO heterostructure (ZC) were designed, and the sensing mechanism was investigated. Experiments show that the ZC sensor has excellent selectivity and sensitivity for HCHO detection at room temperature (RT). Compared with ZnO, there are more amino (NH_2_) groups and hydroxyl (-OH) groups on the surface of ZC, which facilitate to capture more HCHO molecules. Furthermore, there are more oxygen free radicals on the surface of ZC, which can react with absorbed HCHO molecules to improve the performance of the ZC sensor.

## 2. Experiments 

### 2.1. Chemicals and Reagents

Zinc acetate (99%, (CH_3_COO)_2_Zn·2H_2_O) was purchased from Beilian (Tianjin, China, www.Beilian.Chemcp.com). Cupric acetate monohydrate (99%, Cu(CH_3_COO)_2_·H_2_O) was purchased from Guangfu (Tianjin, China, www.guangfu-chem.com). Sodium hydroxide (99%, NaOH) was purchased from Sangon Biotech (Shanghai, China, www.sangon.com). Hexadecyl trimethyl ammonium Bromide (99%, CTAB) was purchased from Aladdin (Shanghai, China, www.aladdin-e.com). All reagents were analytical grade (AR) and can be used directly in experiments.

### 2.2. Synthesis of ZnO and ZnO/CuO Heterostructure

ZnO and ZnO/CuO heterostructures were synthesized by the sol-gel method. Firstly, 2.1 g (CH_3_COO)_2_Zn·2H_2_O and 0.5 g CTAB were dissolved in 100 mL deionized water (DI) and stirred for 5 min at RT. Then, copper acetate was added into the above solution to acquire a uniform blue sol. A certain amount of 0.5 M NaOH solution was dropped into the blue sol, and the pH of the solution was adjusted to 10 by magnetic stirring at RT. After resting at RT for 48 h, the blue gel was heated in a constant temperature oven at 60 °C for 2 h to produce powder. Finally, the powders were annealed in a chemical vapor deposition (CVD) quartz tube furnace at 400 °C for 5 h to obtain ZnO, ZC1, ZC2 and ZC3 samples. The molar ratios of Cu^2+^ to Zn^2+^ in ZnO, ZC1, ZC2 and ZC3 are 0 mol%, 10 mol%, 30 mol% and 50 mol%, respectively. 

### 2.3. Gas Sensing Device Preparation and Measurement

Firstly, the prepared ZnO/CuO powder and DI were mixed into paste. Then, the paste was applied to an Ag–Pd electrode. Secondly, the Ag–Pd electrode was dried at 60 °C for 10 min to form a sensor. Finally, the prepared sensor was aged at RT for 48 h. The gas sensitivity was tested by electrochemical workstation of type CIMPS-2 (ZAHNER ENNIUM, Kronach, Germany) at RT and the voltage was 4 V.

### 2.4. Characterization

A field scanning electron microscope (FESEM, Hitachi, SU8010, Tokyo, Japan) was used to examine the surface morphology of the samples. A transmission electron microscope (TEM, Hitachi, JEM-2100F, Tokyo, Japan) was used to record the crystal structure of samples. A diffractometer with Cu-Κα radiation (Bruker AXS Gmbh, D8 Advance, Karlsruhe, Germany) was used to measure X-ray powder diffraction (XRD) patterns of sample. A fourier transform infrared (FTIR) spectrometer (Bruker, Vertex 70, Karlsruhe, Germany) was used to examine surface properties of the samples. A UV-vis spectrometer (PerkinElmer, Lambda 650, Waltham, MA, USA) was used to record the absorbance spectrum of the samples. An X-ray photoelectron spectrometer (XPS, Thermo Fisher Scientific, ESCALAB 250Xi, Waltham, MA, USA) with 1486.6 eV excitation was use to analyzed the elemental compositions of the samples. An electrochemical workstation (ZAHNER ENNIUM, CIMPS-2, Kronach, Germany) was used to measure the I-V curves of the samples.

## 3. Results and Discussions

### 3.1. Characterization of ZnO and ZC 

**Figure 1:**
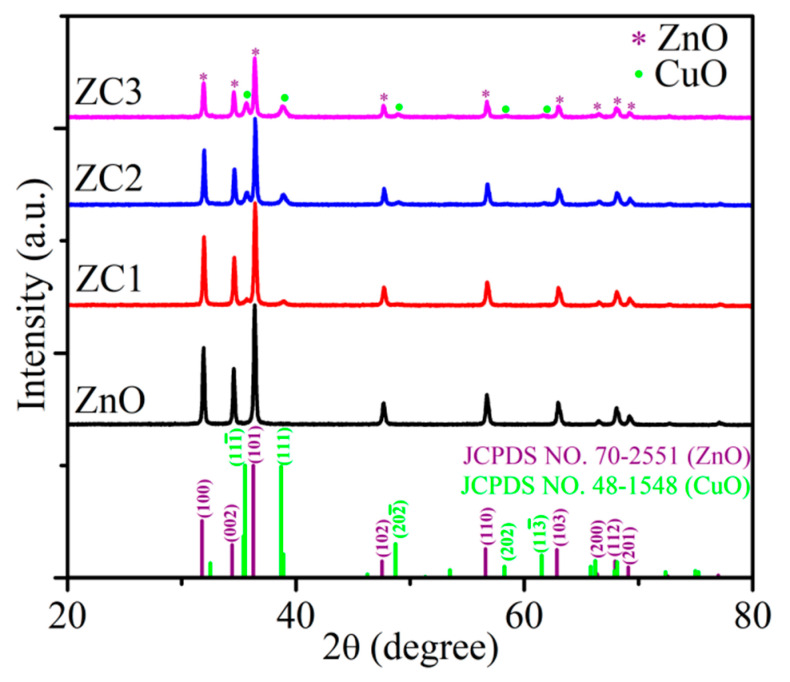
XRD patterns of ZnO and ZC.

XRD patterns of ZnO and ZC are shown in Figure 1. The diffraction peaks at 31.776°, 34.419°, 36.260°, 47.543°, 56.610°, 62.858°, 66.394°, 67.959°, and 69.103° correspond to (100), (002), (101), (102), (110), (103), (220), (112), and (201) faces of the wurtzite structure of ZnO (JCPDS No. 70-2551), respectively. The consistent diffraction peaks of ZnO and ZC indicated that the lattice mismatch between ZnO and ZC was relatively small. Diffraction peaks of 35.543°, 38.708°, 48.716°, and 61.524° for all ZC samples correspond to (11-1), (111), (20-2) and (11-3) faces of monoclinic of CuO (JCPDS No. 48-1548). In addition, the intensity of the diffraction peak for CuO was enhanced gradually with the increase of Cu^2+^ content, and no other impurity peaks were found in the XRD spectra of CuO/ZnO.

**Figure 2:**
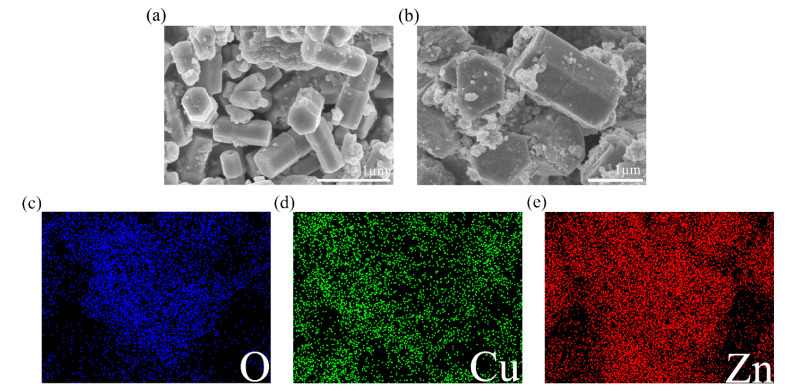
FESEM images of (**a**) ZnO and (**b**) ZC2. Elemental color mapping of ZC2 for (**c**) O, (**d**) Cu, (**e**) Zn.

Surface morphology of ZnO and ZC2 was studied by scanning electron microscopy (SEM). As shown in Figure 2a, ZnO appears as hexagonal prisms with a typical side length of about 300 nm and a height of about 770 nm. In Figure 2b, ZC2 appears as rod-like structures with typical diameters of 1.00–1.21 µm. It can be seen that some particles with a diameter of 30–100 nm are aggregated on the surface of ZC2, which increases the surface roughness and is beneficial to the reaction between ZC2 and the target molecules. The energy dispersive X-ray (EDX) element analyses of ZC2 were carried out by a color chart analysis method. Figure 2c–e shows the element mapping of O, Cu and Zn, respectively. This indicates that Zn, Cu and O are uniformly distributed on the surface of ZC2. 

**Figure 3:**
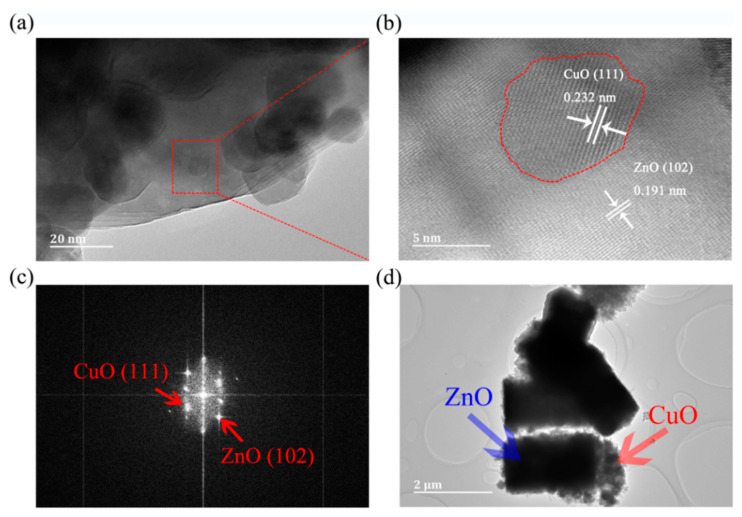
(**a**) HRTEM image of ZC2. (**b**) A magnified view of the enclosed regions in (**a**). (**c**) Fast Fourier transform (FFT) of (**b**). (**d**) TEM image of ZC2.

Figure 3a shows that CuO particles are loaded on the surface of ZnO. Figure 3b shows a typical HRTEM image of a selected region in ZC2 corresponding to Figure 3a. The lattice fringes are in different directions, and lattice spacing distances are 0.232 nm and 0.191 nm, which correspond to the (111) plane of CuO and (102) plane of ZnO, respectively. In addition, the interface between ZnO and CuO particles is represented by a red dotted line, and stable ZnO and CuO lattice fringes can also be found in Figure 3b, which indicates that the structure of ZnO and CuO in P-CuO/N-ZnO gas sensors is a heterojunction. Figure 3c shows the fast Fourier transform (FFT) of Figure 3b. According to the FFT diagram, the crystal plane can be further determined as a ZnO (102) and CuO (111) lattice plane [1,26]. Figure 3d shows that ZC2 has a diameter of 1.00–1.21 µm and a height of 1.99–2.30 µm with small CuO particles loaded on ZnO, which is consistent with that of the SEM in Figure 2b. 

**Figure 4:**
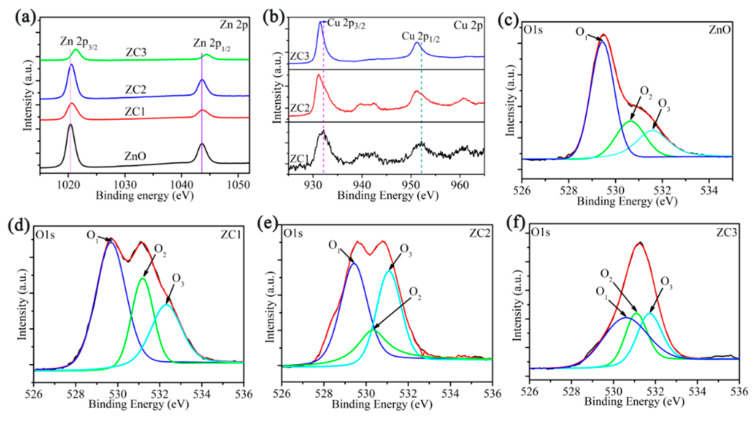
(**a**) XPS spectra of Zn 2p for ZnO and ZC. (**b**) XPS spectra of Cu 2p for ZC. (**c**–**f**) XPS spectra of O 1s for ZnO, ZC1, ZC2 and ZC3, respectively.

XPS analyses of ZnO, ZC1, ZC2 and ZC3 are shown in Figure 4. In Figure 4a, the XPS peaks of Zn 2p_1/2_ and Zn 2p_3/2_ in ZnO are at 1020.36 eV and 1043.45 eV, respectively. The Zn ion is in the oxidation state of +2, due to the difference of the two energy levels of Zn 2p being 23.09 eV [35]. Moreover, compared with the Zn 2p peak of ZnO, the Zn 2p peak of ZC moves towards the direction of greater binding energy, and results in a strong interaction between CuO and ZnO [18]. Figure 4b shows that the binding energies of Cu 2p_1/2_ and Cu 2p_3/2_ are 952.15 eV and 932.17 eV, respectively. Thus, the chemical valence of the Cu ion is +2 due to the difference of binding energies of Cu 2p_1/2_ and Cu 2p_3__/__2_ being 20.02eV [36]. At same time, peaks of Cu 2p shift to the direction of low binding energy with increasing CO in ZC, which is attributed to the large electron shielding effect of the Cu^2+^ ion [37]. Figure 4c–f shows that the O1s peak in ZnO, and ZC can be decomposed into lattice oxygen in the ZnO crystal (O_1_), oxygen vacancy or oxygen defect on the surface of sample surface (O_2_), and surface free oxygen (O_3_) including O_ads_^−^, O_ads_^2−^, O_2ads_^−^. The O_2_ contents of ZnO, ZC1, ZC2, and ZC3 are 27.10%, 36.24%, 25.70%, and 26.67%, respectively. O_3_ contents of ZnO, ZC1, ZC2, and ZC3 are 21.60%, 25.13%, 32.90%, and 27.89%, respectively. It should be noted that ZC2 has the most O_3_ content, which can react with more HCHO gas. 

**Figure 5:**
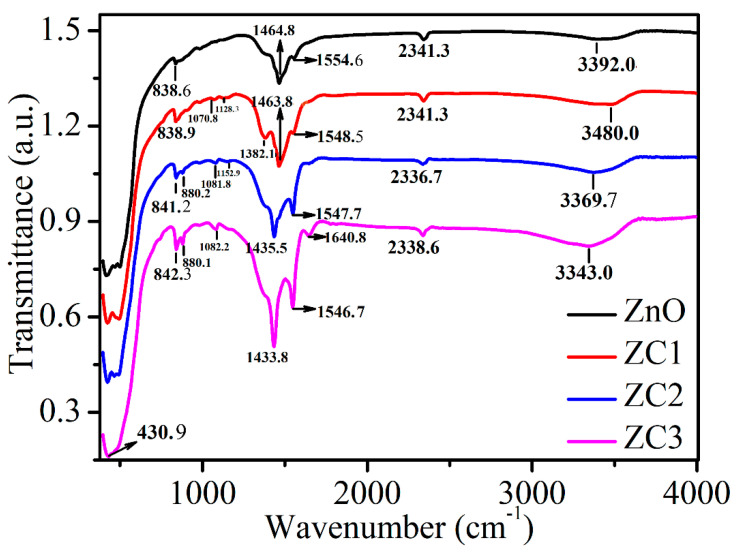
FTIR spectra of ZnO, ZC1, ZC2 and ZC3.

Figure 5 shows the FTIR spectra of ZnO, ZC1, ZC2, and ZC3. The absorption peak at 3200–3600 cm^−1^ corresponds to the stretching vibrational bonds of the hydroxyl group (OH^−^) in the samples [35], and its intensity increases with the increase of CuO content. The peak at around 2341.4 cm^−1^ is caused by the CO_2_ molecule in the air, and the peak at 1640.8 cm^−1^ results in C–H and C=C [38,39]. The peaks at 1554.6, 1548.5, 1547.7, and 1546.7 cm^−1^ correspond to the in-plane bending vibrations of the amino group (N–H) [40], which are increased significantly with the increase in CuO content. The peaks at 1464.803, 1463.9, 1435.5, and 1433.8 cm^−1^ correspond to the in-plane bending vibrations of the methyl group (CH_3_) [41]. The peaks at 1382.1 and around 838.6 cm^−1^ are in-plane bending vibrations of C–H [42,43]. Absorption peaks in the range of 900–1300 cm^−1^ can be attributed to stretching bands of C–C, C–N and C–O [43,44]. The strong absorption peaks in 400 cm^−1^ to 510 cm^−1^ are related to the combination of Cu–O and Zn–O bond vibrations [42]. 

### 3.2. Sensor Testing 

**Figure 6:**
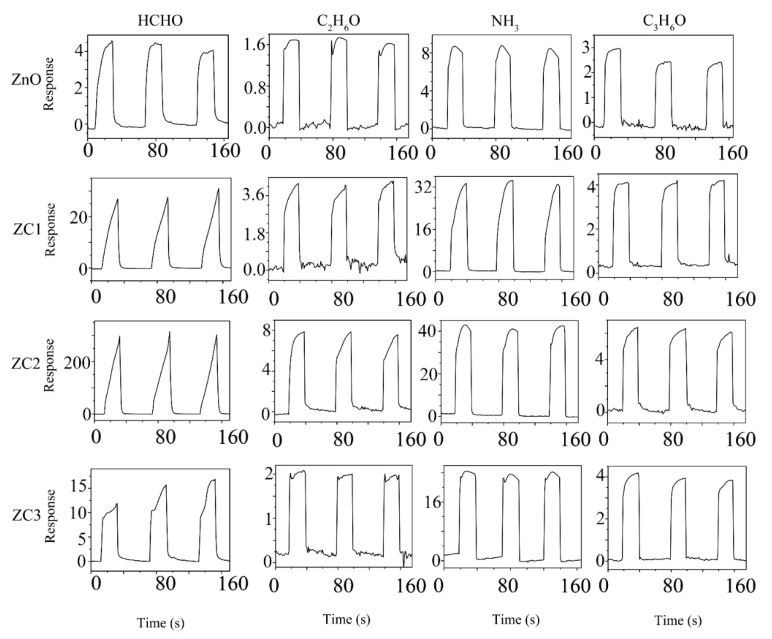
Response curves of ZC with different Cu^2+^ content to 1000 ppm HCHO, C_2_H_6_O, NH_3_ and C_3_H_6_O vapors at room temperature.

Figure 6 shows three successive cycles of response curves of ZnO and ZC sensors when used to detect 1000 ppm target gases of HCHO, C_2_H_6_O, NH_3_, or C_3_H_6_O at RT. The sensing response can be identified as ΔI/I_r_ = (I_r_ − I_g_)/I_r_, where I_g_ is the current of the sensor in the target gas and I_r_ is the current of the sensor in the reference gas. Air is the reference gas in this experiment. In Figure 6, the current of the sensor rises rapidly when the sensor is placed in HCHO, C_2_H_6_O, NH_3_, or C_3_H_6_O vapor, which indicates that ZC and ZnO are n-type semiconductor sensors. Compared with ZnO, the response of the ZC sensor increases with the addition of CuO, which proves that CuO and ZnO heterojunction structures can enhance the sensing performance of ZnO. Among all the gas tests, ZC2 had the largest response to HCHO at a concentration of 1000 ppm, with a response value of about 16,200.

**Figure 7:**
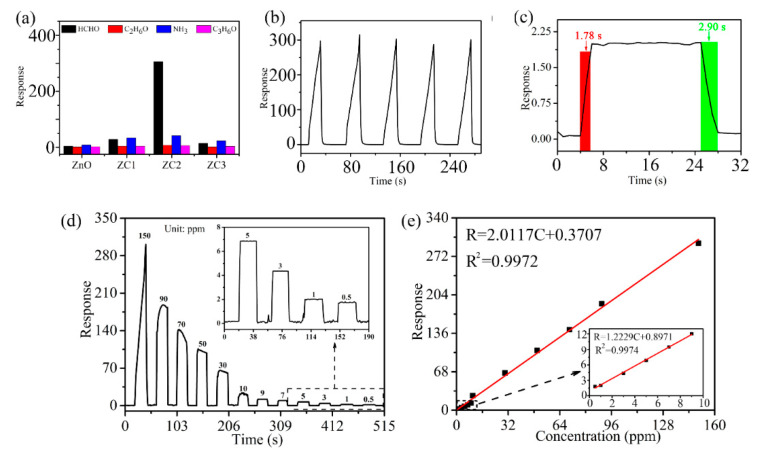
(**a**) Average peak responses and standard deviations for three consecutive cycles of different target gases at 1000 ppm at RT. (**b**) Reproducibility test for 1000 ppm HCHO. (**c**) Dynamic response curves of ZC2 sensor to detect HCHO with concentrations of 0.5–90 ppm, where the insert is an amplification of low-concentration responses. (**d**) Fitting results of response vs. concentration of (**c**). (**e**) Dynamic response–recovery curve of ZC2 sensor to 1 ppm HCHO. The inset shows the data for the low concentration region (<10 ppm).

Compared with other samples, statistical data in Figure 7a shows that the ZC2 sensor has the highest response to HCHO, which means the ZC2 sensor has good selectivity for HCHO gas. In Figure 7b, the ZC2 sensor is used to test 1000 ppm HCHO for four consecutive cycles, which indicates that the ZC2 sensor has good repeatability. Figure 7c shows that the response of the ZC2 is improved with the increase in HCHO concentration from 0.5 ppm to 90 ppm. Figure 7d shows the linear relationship corresponding to Figure 7c for low HCHO concentrations, and the fitting results are R = 1.22291 C + 0.89707 and R^2^ = 0.9974. It is worth noting that the slope is larger at lower concentrations, which means that the response increases faster at a low HCHO concentration. The detection limit is defined as LOD = 3SD/m, where m is the slope of the linear part of the calibration curve and SD is the standard deviation of the noise in the response curve [35]. In this experiment, the LOD of ZC2 for HCHO is calculated to be 0.079 ppm, according to fitting results.

Response time and recovery time are closely related to the detection speed of sensors, which are defined as the time required from the sensor contacting with the measured gas to reaching 90% of the stable value, and the sensor leaving the measured gas to returning to 10% of the stable value, respectively. Figure 7e shows that the response/recovery time of the ZC2 sensor are 1.78 s/2.90 s when it is exposed to 1 ppm HCHO at room temperature. Table 1 lists the performance parameters of HCHO sensors reported in recent literatures and the HCHO sensors based on ZC2 in this work. The response/recovery time of the ZC2 sensor is shorter compared with the reported HCHO sensors, except for the copper (II) complex that has a slightly lower detection limit, but significantly longer times [31]. Therefore, HCHO based on the ZC heterojunction in this work presents a lower detection limit and a fast response/recovery time. 

**Figure 8:**
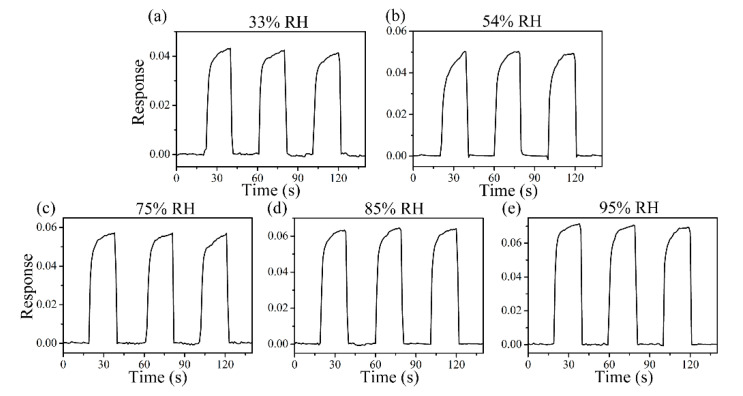
Response curves of ZC2 sensor to 33%, 54%, 75%, 85% and 95% RH at RT.

Figure 8 shows the response curve of the ZC2 sensor in different humidity environments, which are used to test the tolerance of the ZC2 sensor to relative humidity (RH). The different humidity environments of 95%, 85%, 75%, 54%, 33%, and 11% RH were controlled by super saturated aqueous solutions of KNO_3_, KCl, NaCl, Mg(NO_3_)_2_, MgCl_2_, and LiCl, respectively. The variation in response can be measured by ΔI/I = ΔI/I**_b_** = (I_RH_ − I_b_)/I_b_, where I_b_ is the base response at 11% RH at room temperature and I_RH_ is the current value tested in the target humidity [35]. Figure 8 shows that the relative changes of ZC2 for 33%, 54%, 75%, 85%, and 95% RH are 0.042, 0.050, 0.057, 0.064, and 0.071, respectively, which indicates that the ZC2 sensor has excellent resistance to humidity. 

FTIR results show that more OH– and N–H groups appeared on the surface of ZC2. These hydrophilic functional groups facilitate the absorption of water molecules, but the ZC2 has excellent resistance to humidity. This is because the amount of oxygen vacancies in ZnO and ZC2 heterojunctions is almost identical in XPS analysis. In humidity detection, oxygen vacancies determine the decomposition of water molecules when water molecules are adsorbed on the surface of the material. 

**Figure 9:**
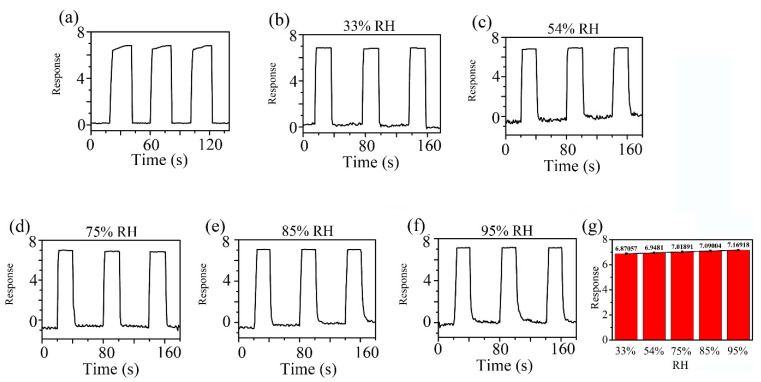
Response curves of ZC2 sensor to 5 ppm HCHO gas (**a**) in desiccant, (**b**–**f**) under different humidity environments at room temperature. (**g**) The average values of the responses in different RH.

Figure 9 indicates the dynamic response profiles of 5 ppm HCHO gas in different humidity environments at RT. In Figure 9a, the response of the ZC2 sensor to 5 ppm HCHO containing desiccant is 6.81. Figure 9b–f shows that the responses of ZC2 sensor to 5 ppm HCHO at 33%, 54%, 75%, 85%, and 95% RH are 6.87, 6.95, 7.02, 7.09, and 7.17, respectively, which means humidity in the environment has little effect on HCHO detection based on ZC2.

### 3.3. Sensing Mechanism

**Figure 10:**
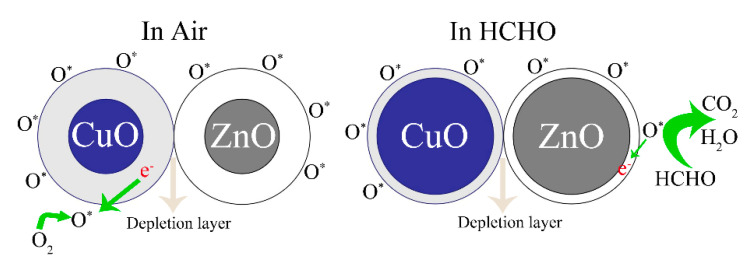
Mechanism diagrams of ZC sensor in the air and HCHO.

Figure 10 shows the sensing mechanisms of the ZC sensor in the air and HCHO gas. In the ZnO/CuO heterostructure, electrons move from the ZnO n-type semiconductor to the CuO p-type semiconductor. Electrons move from ZnO to CuO in the ZC heterostructure due to the higher Fermi level of ZnO, and holes move from CuO to ZnO until the Fermi level reaches equilibrium. This phenomenon inhibits the recombination of holes and electrons, making more free electrons exist on the surface of ZC. More oxygen molecules are attached to surface of the sensor when the semiconductor sensor is placed in the air. At the same time, oxygen atoms capture free electrons to form free oxygen O*, forming a large electron depletion layer that increases the resistance and reduces the current. A large number of HCHO molecules are adsorbed on the surface of CZ when the sensor is placed in HCHO gas, which is due to the fact that -OH and NH_2_ groups have strong absorbance ability to HCHO. Then, the adsorbed HCHO molecules react with more free oxygen (O*) to form CO_2_, H_2_O, and e^−^. The reaction equations are given below:

O_2gas_ → O_2ads_ (1)

O_2ads_ + e^−^ → O_2ads_^−^ (2)

O_2ads_^−^ + e^−^ → 2O_ads_^−^ (3)

HCHO_gas_ → HCHO_ads_ (4)

HCHO_ads_ + O_2ads_^−^ → CO_2_ + H_2_O + e^−^ (5)

HCHO_ads_ + 2O_ads_^−^ → CO_2_ + H_2_O + 2e^−^ (6)

The released electrons return to the semiconductor sensor, which makes the electron depletion layer become thinner, the resistance decrease, and the current increase.

## 4. Conclusions

In summary, HCHO sensors with high performance based on ZC were designed and the sensing mechanism was investigated. Experiments show that there are more free oxygen radicals, N–H groups, and OH^−^ groups on the surface of ZC with an increase in Cu^2+^ content. Furthermore, ZC2 has a short response/recovery time (1.78 s/2.90 s) when HCHO concentration is 1 ppm, and the LOD of ZC is 0.079 ppm. Moreover, ZC has good resistance to humidity interference, which indicates that ZC has a potential in the application of HCHO sensors with high performance.

## Figures and Tables

**Table 1:** Comparison of ZC (ZnO/CuO heterostructure) and other material sensors in recent reports.

Materials	Temperature (°C)	LOD (ppm)	Response/Recovery Time (S)	Concentration (ppm)	References
MIL-101(Cr)	25	1.794	<15/<58	2	[45]
PODS-PDA	25	1	11/6	1	[46]
copper (II) complex	25	0.05	9 /11	0.05	[30]
ZnCo_2_O_4_	180	-	9/12	100	[47]
PdO-ZZCO	139	0.2	9/14	100	[48]
Pt-decorated MoO_3_ nanowires	25	1	17.8/10.5	100	[49]
Hollow TiO_2_@SnO_2_ nanospherns	25 (UV-LED)	-	20/56	10	[50]
ZnO/CuO heterostructure	25	0.079	1.78/2.90	1	This work

## Data Availability

Data available in a publicly accessible repository.
